# Variations in Obesity Rates between US Counties: Impacts of Activity Access, Food Environments, and Settlement Patterns

**DOI:** 10.3390/ijerph14091023

**Published:** 2017-09-07

**Authors:** Peter Congdon

**Affiliations:** School of Geography, Queen Mary University of London, London E1 4NS, UK; p.congdon@qmul.ac.uk

**Keywords:** obesity, poverty effect, Bayesian regression, urban-rural, food environment, inactivity, commuting, spatial clustering

## Abstract

There is much ongoing research about the effect of the urban environment as compared with individual behaviour on growing obesity levels, including food environment, settlement patterns (e.g., sprawl, walkability, commuting patterns), and activity access. This paper considers obesity variations between US counties, and delineates the main dimensions of geographic variation in obesity between counties: by urban-rural status, by region, by area poverty status, and by majority ethnic group. Available measures of activity access, food environment, and settlement patterns are then assessed in terms of how far they can account for geographic variation. A county level regression analysis uses a Bayesian methodology that controls for spatial correlation in unmeasured area risk factors. It is found that environmental measures do play a significant role in explaining geographic contrasts in obesity.

## 1. Introduction

Upward trends in overweight and obesity rates are a major public health concern, with recent US estimates showing over a third of adults to be obese [1]. Obesity increases the risk of heart disease, stroke, type 2 diabetes, and certain cancers, and increased obesity has been linked to recent slowing of improvements in heart disease mortality in the US [2]. Reduced levels of physical activity, increased sedentary behaviour, and changing dietary behaviours (e.g., more consumption of processed food with high sugar and fat content) have been identified as primary factors in upward obesity trends [3,4].

### 1.1. Urban Environment and Obesity

There is much ongoing research about the effect of environment and place of residence as against individual behaviour on growing obesity levels [5,6,7]. Recent evidence suggests that development of excess weight through individual characteristics (e.g., low income) or adverse health behaviours (e.g., physical inactivity) can be mediated by environmental influences and neighbourhood socioeconomic status [8,9,10,11]. There is also strong evidence of contextual variation in obesity prevalence: with regard to the US, Drewnoski et al. [12] report greater disparities in obesity rates by ZIP code area than those associated with individual income or race/ethnicity. Contextual variation refers purely to place effects on geographic health contrasts as distinct from impacts due to the population composition of different areas; for example [13] set out principles useful for understanding how contextual characteristics can affect health and health disparities. 

A further aspect of geographic obesity variation is high spatial clustering of obesity rates [14,15]. Spatial clustering in obesity reflects a similar spatial clustering in geographic factors (e.g., food environment, urban configuration, exercise access) that affect obesity. Some of these factors can be measured, but there also will be unmeasured (i.e., unobserved) geographic influences on obesity that will also tend to be spatially clustered. Such spatial effects proxy unobserved risk factors (e.g., environmental or cultural) which “typically vary smoothly over space” ([16], p. 195). Not controlling for spatially correlated residual variation may lead to overestimated statistical significance of regression effects for known place variables [16,17].

Various behavioral and environmental aspects have figured in recent epidemiological literature. Changing exercise levels have been cited as a major reason for increased obesity [18,19], and so access to exercise opportunities is relevant [6]. Thus [18] report that “body mass index and waist circumference trends were associated with physical activity level but not daily caloric intake”. On the other hand, some studies (e.g., [20]) stress changing dietary patterns, and the local food environment is accordingly a major research focus [21,22]. There is extensive research documenting variation in access to healthy foods (fresh fruit, vegetables, and other whole foods), with lower access tending to be found in socio-economically deprived areas or ethnic minority neighbourhoods [23,24]. This is taken to reflect the location of supermarkets, grocery stores, and farmers’ markets, as opposed to fast food outlets and convenience stores offering processed food with high sugar and fat content. 

Impacts of the environment on weight levels depend to a considerable extent on the national context. For example [25], European countries with comparatively high rates of walking and cycling have less obesity than do the US, Canada, and Australia which are more car dependent. Similarly, sprawl patterns characteristic of US suburban development are not necessarily replicated in European cities. Nevertheless, changes in food consumption patterns [26] and reductions in activity levels [19] are common to many countries, both developed and developing.

Especially in the US, changing activity patterns and limitations on healthy food access have been linked to sprawl [27,28,29], namely dispersed low density post-WW2 suburban development, with segregated land uses, low walkability [30], and high automobile dependence. Sprawl tends to lead to more driving because of extended distances to workplaces or facilities, and because urban design and environment (e.g., land use mix, street connectivity, housing density) affect the likelihood of using active transport modes for commuting and other trips [31]. By contrast, walkable neighbourhoods facilitate walking or bicycling to amenities such as shopping centres, parks, schools, and entertainment centres, rather than requiring automobile trips. Car commuting and time spent in car travel (as compared with active travel modes) have themselves been linked to obesity risk [32,33,34,35].

### 1.2. Contribution of This Study

A number of studies have focused particularly on geographic variations in obesity and the impacts of neighborhood measures of physical activity, healthy food access, or urban dispersal [36,37,38,39,40]. Existing studies may have a restricted geographic focus; may consider relatively historic data; may not allow for residual spatial correlation; or have not necessarily jointly considered impacts of activity access, food environment, and urban dispersal patterns. This paper is distinctive in providing a comprehensive approach to geographic dimensions of obesity variation in the US, using recent data and a national perspective, and in the comprehensive range of environmental factors used to account for such variation. It is also distinctive in assessing how far environmental factors explain spatial clustering in obesity.

The paper considers obesity variations between US counties, using county obesity prevalence rates for 2013. These are age standardized and provided by the Center for Disease Control and Prevention [41] for persons, males, and females, and for the 50 US states and the District of Columbia, but excluding Puerto Rico. They are based on Behavioral Risk Factor Surveillance System (BRFSS), an annual cell phone survey. Below, we delineate the main dimensions of geographic variation in these obesity rates between counties: by the urban-rural status of counties, by their region of location, by area poverty status, and by majority ethnic group (see Section 1.3).

We subsequently use regression methods (see Section 2 and Section 3) and incorporate seven available measures of activity access, food environment, and settlement patterns to assess how far they can account for geographic variation in obesity. County indicators of exercise access are leisure time inactivity rates and an index of access to exercise opportunities [42], measured by closeness to locations for physical activity. As indicators of food environment, we take the following: percentage of all restaurants that are fast-food establishments; grocery stores per head of population; and the ratio of convenience stores to groceries. An additional indicator is provided by the Food Environment Index [43]. This index ranges from 0 (worst environments) to 10 (best environments), and equally weights two indicators of the food environment: limited access to healthy foods and food insecurity. To provide an indicator of dispersed car dependent settlement, we obtain a principal component score based on car as against active commuting, post 1950-housing, and population density [44,45].

The county level analysis uses a Bayesian methodology to estimate linear regression and specify spatial clustering in unmeasured area risk factors. The latter is an important feature given that it is not feasible to measure all relevant aspects of the obesogenic environment. Under Bayesian inference [46], parameters are considered as random variables, with initial knowledge about parameters represented by a prior distribution, and updated knowledge (taking account of actual data) represented by a posterior distribution. Despite limitations in the available measures of environment [9], it is found that such measures do play a significant role in explaining geographic contrasts in obesity. This study thus provides a new use of existing data, develops a new index of commuting/settlement and shows its utility in explaining obesity variations, and investigates the utility of the recently developed Food Environment Index in accounting for obesity contrasts.

### 1.3. Geographical Dimensions of Obesity Variation in the US

**Figure 1:**
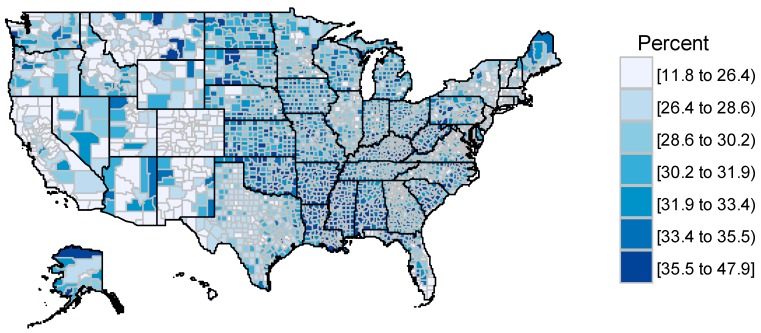
US county obesity rates (persons, 2013).

To set out full the background to the study, we consider the finer detail of geographic variation in obesity that we later seek to explain. Geographic contrasts show first in regional differences: higher obesity in southern US regions, particularly the south central states such as Arkansas, Mississippi, Alabama and Tennessee (see Figure 1, mapping county obesity rates for all persons, with black lines for state boundaries). Such regional variations overlap with contrasts by area poverty status [47,48] and urban-rural status.

To demonstrate the interplay between poverty and urban-rural status, Table 1 shows the wide variations in obesity rates associated with different geographic settings, as defined by urbanicity and area socioeconomic status. For females, the most extreme contrast is an obesity rate of 26% in low poverty highly metropolitan counties as against 36% in high poverty counties either completely rural or with under 2500 urban population, but adjacent to a metro area.

Population ethnic/race composition is also a significant source of geographic variation in obesity. The Center for Disease Control and Prevention (https://www.cdc.gov/obesity/data/prevalence-maps.html) reports that non-Hispanic blacks had the highest prevalence (over 2013–2015) of self-reported obesity (38.1%), followed by Hispanics (31.9%), and non-Hispanic whites (27.6%). Translated into geographic contrasts, for non-Hispanic white adults, no states had an obesity prevalence of 35% or greater, whereas for non-Hispanic black adults, 28 states and the District of Columbia obesity rates of 35% or more. There is also evidence of excess obesity among American Indian or Alaska native groups. Growth in obesity rates among these communities and increases in associated diseases, such as diabetes, have been documented [49], with the US Office of Minority Health reporting (https://minorityhealth.hhs.gov/omh) an age-adjusted obesity rate (in 2014) for American Indian/Alaska Native adults of 42.3%, compared with 27.4% among white non-Hispanics.

In the present analysis, we use the census division a regional descriptor. These are subdivisions of four US census regions—namely the Northeast, Midwest, South, and West—with each of the four regions then divided into two or more Census divisions. As a measure of urban-rural status we use the urban-rural classification scheme for counties prepared by the National Center for Health Statistics (NCHS) [50]. For poverty status, we disaggregate counties into poverty quintiles, and to summarise variations in ethnic population, we characterise counties according to the majority ethnic group in the county (white non-Hispanic, black non-Hispanic, Hispanic, or other).

**Figure 2:**
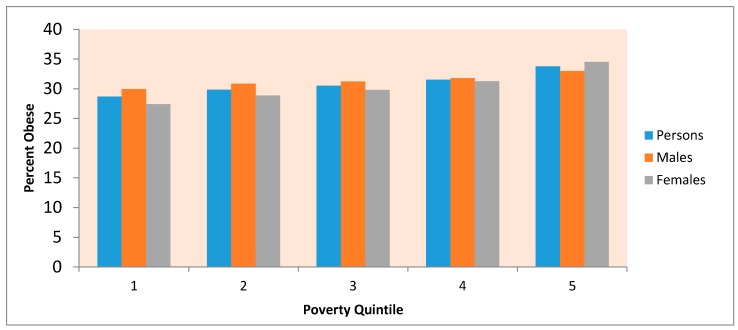
Obesity gradient according to county poverty quintile *. *: 1. 20% of Counties with lowest poverty (under 11.8%); 2. Counties with poverty rates between 11.8% and 14.7%; 3. Counties with poverty rates between 14.7% and 18%; 4. Counties with poverty rates between 18% and 22.1%; 5. 20% of counties with highest poverty (over 22.1%).

The rise in obesity as poverty increases (rightmost column, Table 1) accords with research showing socioeconomic gradients in obesity in the US; for example [51] mentions that “there is no question that the rates of obesity and type 2 diabetes in the United States follow a socioeconomic gradient”, meaning that each successively more advantaged group has lower diabetes and obesity rates; see also [52] for discussion of social gradients in health. Focusing on the poverty gradient in obesity rates, Figure 2 shows a steeper gradient among females than males (i.e., a more marked contrast in obesity between low and high poverty areas), a feature discussed in the literature (e.g., [53]). This shows most simply in the ratio of rates in the highest to lowest poverty counties, 1.26 for females as against 1.10 for males.

Some of the impacts of ethnicity may be mediated by differing socio-economic status and poverty levels. Table 2 accordingly cross-tabulates obesity rates by area poverty status and the county majority ethnic/race group, and shows how these two factors act together to affect obesity rates. Thus the highest obesity rates (42%) are for females in high poverty counties with a majority black non-Hispanic, as against 27% female obesity in low poverty areas with a majority white non-Hispanic. 

Finally Table 3 shows obesity contrasts by US Census division and poverty status. As in Table 1, contrasts are greater for females: 23% in low poverty New England counties as against 38% in high poverty counties in East South Central (Alabama, Kentucky, Mississippi, and Tennessee). Contrasts between census divisions regardless of poverty status (in the ‘All counties’ row) are greater for females, by over 10 percentage points: 35.5% in East South Central compared with 25% in New England. This compares with the widest male contrast, by seven percentage points: 34.1% in East South Central compared with 27% in New England.

## 2. Methods: Bayesian Regression Analysis of US County Obesity Rates

### 2.1. Regression Methods

Two regression analyses are carried out, involving linear regression with obesity rates as the outcome. First, in Regression 1 (Geographic Categories Only) we demonstrate the impact on obesity of geographic categories (urban-rural, census division, county poverty status, county majority ethnicity/race) using only these four categorisations (and the corresponding six first order interactions) as predictors. First order interactions are modelled as random effects. The analysis is carried out in the WINBUGS program [54].

In a second extended regression, Regression 2 (Geographic Categories and Environmental Indicators), we include both the geographic categories and a set of indicators of the obesogenic environment. We aim in the extended regression to assess how far it is possible to explain geographic variability by indicators of exercise access, food environment, and settlement patterns. 

Total residual variation is partitioned between an IID residual (independent and identically distributed), and a spatially correlated residual, following an intrinsic autoregressive form, implemented using the car.normal option in WINBUGS [55]. 

Thus denote y_i_ as the obesity rate in county i, and X_i_ as known (i.e., actually observed) area risk factors. Furthermore let s_i_ denote the county residual effect which represents spatial clustering in obesity, and u_i_ denote as normally distributed IID residual, u_i_ ∼ N(0, $\sigma_{u}^{2})$, where N(m,V) represents the normal distribution with mean m and variance V. The s_i_ are conditionally normally distributed with a mean s_i_, defined by the average of the effects s_j_ (j ≠ i) within the subset of L_i_ counties adjacent to county i, and with conditional variance $\sigma_{s}^{2}$/L_i_. Then we assume

Y_i_ = X_i_β + s_i_+ u_i_ (1)

where β are regression coefficients representing the effects of actually measured indicators of the food, exercise, and urban environment. This regression may be represented in hierarchically centred form as y_i_ ∼ N(X_i_β + s_i_, $\sigma_{u}^{2}$).

As a measure of variation explained by the geographic categories, we use 1 − (Total Residual Variation)/(Total Variation). As a measure of spatial clustering in the residual variation, we compare the marginal spatial variance var(s) with the variance $\sigma_{u}^{2}$ of the IID residuals, namely

(2) $$\Lambda =\mathrm{var}(s)/[\mathrm{var}(s)+\sigma_{u}^{2}],$$

with values between 0 and 1, and higher values showing a stronger spatial clustering; see [56], p. 139. 

Inferences are based on Markov Chain Monte Carlo estimation [57], which estimates parameters by drawing repeatedly from joint density of parameters given the observed data (the posterior density). Inferences are from the second half of a two chain run of 10,000 iterations, with convergence assessed by Brooks-Gelman-Rubin (BGR) diagnostic statistics [58]. Results are expressed in terms of posterior means and 95% credible intervals. For a 95% credible interval, the value of interest (e.g., a regression coefficient) is located with a 95% probability in the interval. The analogous statistics from a classical analysis would be point estimates and their 95% confidence intervals, though interpretation differs [46]. 

### 2.2. Methods: Defining Environmental Indicators and their Relevance to Obesity

As mentioned above, we aim to find how far environmental indicators explain geographic obesity variations, and how far they account for spatial patterning in obesity. Seven indicators of the environment are included in the extended regression, and it is important to establish their potential relevance to explaining obesity.

**Figure 3:**
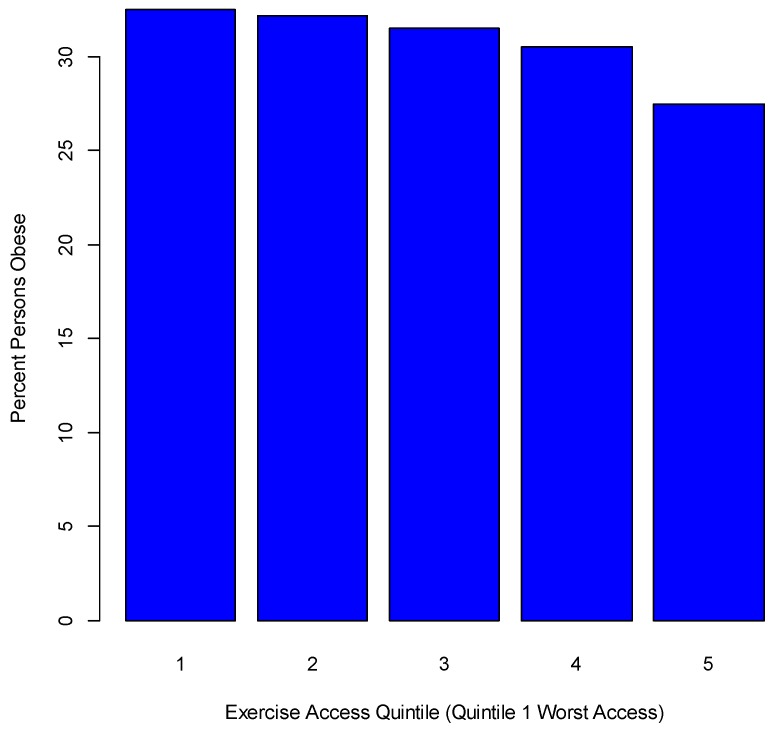
Percentile obesity rate by adequate exercise access.

Regarding exercise levels, two indicators are included in the extended regression. The first is an index of adequate access to exercise opportunities [42], namely the percentage of individuals in a county who live reasonably close to locations for physical activity (parks or recreational facilities). Adequate access is defined as resident in a census block within a half mile (0.805 km) of a park, resident in an urban census block within one mile (1.61 km) of a recreational facility, or resident in a rural census block within three miles (4.83 km) of a recreational facility. The potential relevance of this indicator in explaining obesity variations is apparent if the access index is converted to quintiles: the 20% of counties with the lowest access have an obesity rate of 32.5%, whereas the 20% of counties with the highest access have an obesity rate of 27.5% (see Figure 3). It is of interest that the highest percentages for adequate exercise access are in the most metropolitan counties (NCHS urban-rural category 1). 

Arguably, a more direct indicator of exercise access is the inactivity rate, specifically leisure time inactivity. Average obesity is 37.1% for the 20% of counties with the highest levels of inactivity, compared with a rate of 24.3% in the 20% of counties where inactivity levels are lowest.

**Figure 4:**
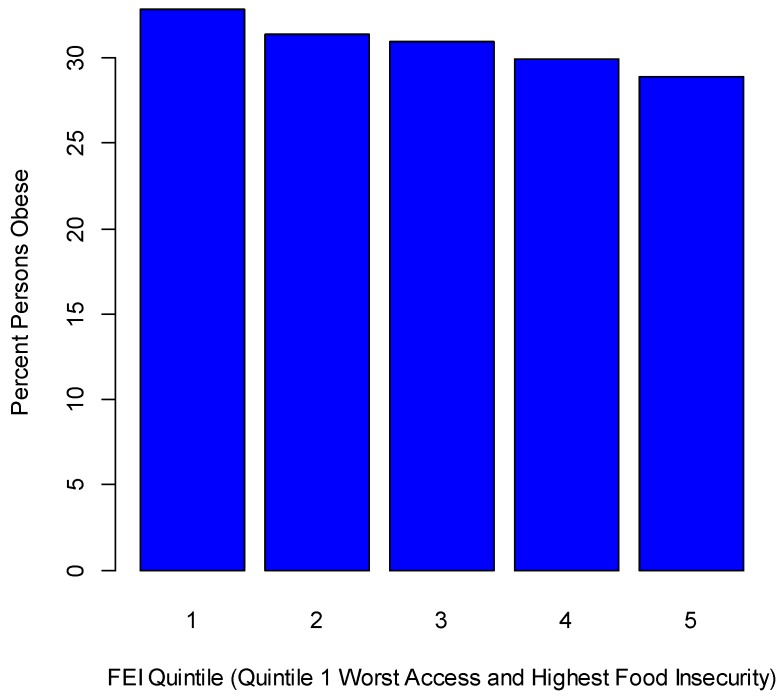
Percentile obesity rate by food environment index (FEI).

As indicators of food environment and food outlet mix, we take the three following: percentage of all restaurants that are fast-food establishments (County Business Patterns); grocery stores per head of population; and the ratio of convenience stores to groceries. The latter two indicators are from the Food Environment Atlas [59] and the first is from the 2013 County Health Rankings [60]. An additional indicator is the Food Environment Index [43], based in turn on the following: (1) limited access to healthy foods, namely the percentage of the population that is low income and does not live close to a grocery store, namely in rural areas, over 10 miles (16.1 km) from a grocery store, and in non-rural areas, over 1 mile (1.61 km); and (2) food insecurity, namely the percentage of the population who did not have access to a reliable source of food during the past year. ‘Low income’ is defined as having an annual family income of less than or equal to 200 percent of the federal poverty threshold for the family size. Links between food insecurity and obesity are well established [61]: thus households with limited resources manage their food budgets by buying cheap, energy-dense foods, typically of lower nutritional quality and, because of high calories, linked to obesity. Furthermore, low income households are less likely to own cars, and car access may in turn affect access to healthy food outlets (e.g., supermarkets). Figure 4 shows obesity rates according to county quintiles on the Food Environment Index (note the highest FEI values, as in quartile 5, denote better access to healthy food and lower food insecurity).

**Figure 5:**
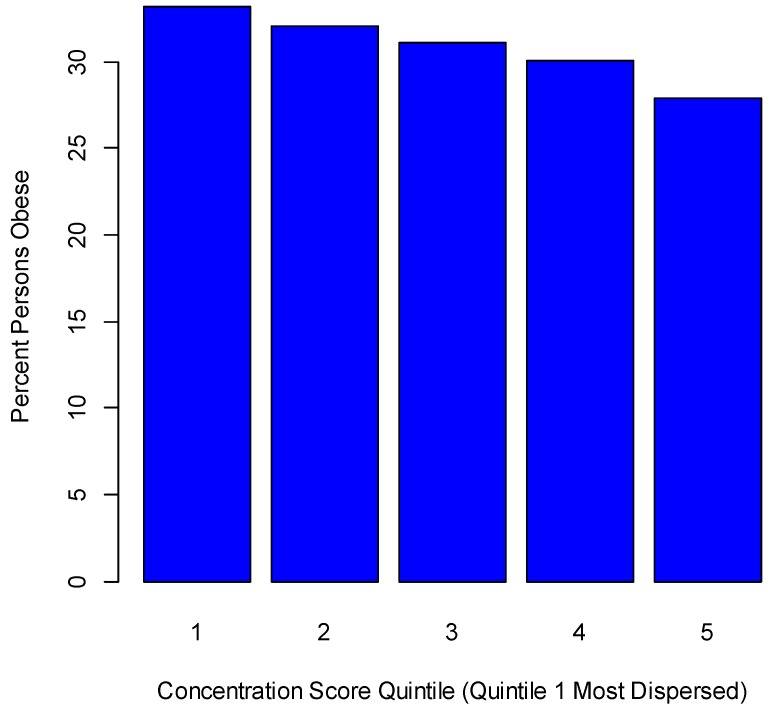
Percentile obesity rate by concentration score quintile.

To provide a general indicator of dispersed car dependent settlement, we obtain a principal component score based on five indices: percentages of car commuting, of public transport commuting, and of walking/cycling commuting; percentage of housing built after 1950; and population density [44,45]. The resulting leading component score loads negatively on car commuting and post-1950 housing, and so is a negative measure of dispersal, with highly negative scores for counties with the most dispersed development. This score correlates 0.77 with the compactness score of [62] over the 993 counties where both scores are available, and is associated with obesity variations. Grouping the scores into quintiles, with Quintile 1 for the most dispersed, highly car-dependent, low density settlements, and Quintile 5 for the least dispersed, higher density areas, the respective all person obesity rates in Quintiles 1 and 5 are 33.2% and 27.9% (see Figure 5). This score (called the concentration score for simplicity) is the final environmental indicator included in the extended regression.

## 3. Results: Environmental Indicators, Geographic Categories, and County Obesity Rates

### 3.1. Regression Using Geographic Categories Only

Table 4 shows the parameter estimates for Regression 1 (Geographic Categories Only). This regression controls for interdependency between geographic categories, so not all contrasts apparent in Table 1, Table 2 and Table 3 are significant when expressed in terms of regression coefficients. Significant variations for obesity are associated with particular regions: higher obesity rates in the East South Central states for all persons, males and females; and higher male obesity also in West North Central, Middle Atlantic, and West South Central states. There is also a significant poverty gradient apparent for all persons obesity. The obesity excess in extreme poverty areas is higher for females, as is the excess for black majority counties. There is also a marked obesity excess in counties where other ethnic groups are in the majority, most apparently for female obesity [49]. These are counties with American Indian or Alaska native majorities. 

For males, excess obesity is also linked to NCHS urban category, including higher obesity in Categories 4, 6, and 8, namely towns adjacent to metropolitan areas. Variations such as this may be linked to differences in active commuting patterns, access to exercise opportunities, and so on, and may be reduced to insignificance when environmental factors are controlled for. 

### 3.2. Extended Regression

Table 5 shows the results of the extended regression, namely Regression 2 (Geographic Categories and Environmental Indicators), including the seven environmental indicators. To enable comparison of the relative impacts of the indicators on obesity, they are all converted to a [0, 1] scale, (x − min(x))/range(x).

Comparison of Regression 2 against Regression 1 shows the following features: (a) considerably increased levels of explained variation when environmental indicators are included, for example, from 38% to 62% for female obesity; (b) a marked reduction in variation linked to area poverty status; (c) a diminution in the effects of county majority ethnic group; (d) main effect urban-rural and regional categories mostly have insignificant effects in Table 5 (i.e., the 95% credible intervals include zero); (e) considerable reduction in variances associated with interactions between geographic categories; and (f) reductions in the proportion of residual variation that are spatially structured/clustered. 

As an exception to the generally reduced significance for regional effects in (d), the Mountain Census division (Idaho, Montana, Wyoming Arizona, Colorado, Nevada, New Mexico, Utah) emerges as having significantly lower obesity after environmental influences are allowed for. Obesity in the East South Central states is no longer significant after allowing for environmental indicators, though for males a significant excess obesity in the West South Central states remains.

Regarding the relative impacts of the environmental indicators, these are mostly significant. Among the highest impacts are the positive effects on obesity of inactivity, with a smaller negative effect of exercise access. There is also a high negative impact on obesity of the concentration score, which is a negative index of settlement dispersal and car dependence. Relative to measures of activity and settlement patterns, impacts of the food environment are smaller, but still mostly significant, with effects in the expected direction, and with impacts tending to be stronger for females. The food environment index (which ranges from 0, for worst environments, to 10, for best environments) has an expected negative effect, and has the strongest impact of the four food environment indicators among females, and also among all persons.

## 4. Discussion

Features (a) to (f) noted in Section 3.2 are important as they show clearly how far geographic differences in obesity can to a large degree be explained by indicators of activity and food environments, and of settlement and commuting patterns. Feature (d) is important since many studies of US obesity differences note major regional contrasts [63,64], and contrasts according to urban-rural status [65,66]; for example [63] refer to “pronounced regional concentrations of obesity prevalence”. Feature (b) is also important, since there are wide obesity contrasts according to area poverty level (see Figure 2). 

Both regional contrasts and poverty effects lose much of their relevance in explaining area obesity differences (in regression terms) when indicators of food and activity environment, and of settlement and commuting patterns, are used as predictors. This does not mean that regional contrasts or poverty gradients in obesity are unimportant per se, but that their regression effects on obesity operates mainly via environmental differences, and direct effects of poverty and region are much diminished once environment is allowed for. Alternatively expressed, the major part of the poverty effect on obesity seems to be explained by the disproportionate exposure to, and burden of, obesogenic environments (i.e., the environmental injustice) experienced by low-income populations, and the impacts of these environments on behavior [67]. For example, inactivity rates of 28.4% in the 20% of counties with highest poverty rates contrast with rates of 21.6% in the lowest poverty quintile, so allowing for inactivity diminishes the direct poverty effect on obesity rates. Similarly, the major part of the regional effects on obesity seems to be explained by the disproportionate exposure to obesogenic environments in certain regions. Thus [64] mention that “spatial clusters of both higher and lower obesity levels [..] are indicative of regional variations in obesogenic environments and associated risk factors”. 

The last feature, (f), reflects the fact that control for environmental influences has accounted for much of the spatial clustering apparent in obesity maps. Spatial clustering in residuals occurs when relevant area predictors, which themselves tend to be geographically clustered, have not been controlled for. Some of these factors are observed, and once they are controlled for, spatial patterning of residuals is reduced. Remaining spatial clustering can be attributed to unknown or unmeasurable risk factors, which also tend to vary smoothly in space [16].

Regarding the relative impacts of the environmental indicators, effects of both activity indicators are significant. Inactivity effects on obesity are pronounced, and greater for females, this being one source of the overall higher level of explained variation (62%) for females as compared with males (50%). Continuing the environmental justice theme, health-promoting recreation resources may be inequitably distributed across sociodemographic groups, and this is one source of activity differences [68]. Other area factors (crime rates, perceived neighborhood safety, etc.) may also impact on activity levels [69]. 

In Table 5, adequate exercise access has a significant negative effect on obesity, but a relatively smaller one compared with the inactivity effect. This may be because much of the impact of exercise access on obesity is mediated by its effect on activity rates (e.g., [70]). Both inactivity and exercise access are correlated with poverty, namely a correlation of 0.48 (over US counties) between poverty and inactivity, and a correlation of −0.38 between exercise access and poverty. So their inclusion in the regression contributes to explaining the much reduced direct poverty effect apparent in Table 5. Inactivity rates also vary according to majority ethnic group, being higher in majority black counties (around 30% as compared with the all counties rate of 25%); this may partly account for diminution in the effects of county majority ethnic group in Table 5 as compared with Table 4. 

There is also a high negative impact on obesity of the concentration score, which is a negative index of settlement dispersal and car dependence. Hence, areas with positive scores (typically metropolitan, higher density areas, with lower car commuting levels and higher active commuting) have lower obesity rates. This impact is greater for males, and is in fact stronger than the effect of inactivity rates, whereas the reverse is true for females. This may be linked to higher car commuting rates and longer car work trips among males [71]. 

Differences in commuting patterns have not figured in the obesity literature as much as food and activity environments, or in the development of sprawl indices. For example, a recent study uses a multivariate factor method to derive a sprawl index without any reference to commuting patterns [72]. Hence, a more general approach to settlement density and commuting patterns, as used in the present paper, may have explanatory value for area obesity studies.

Relative to measures of activity and settlement patterns, impacts of the food environment are smaller, but still mostly significant, with effects in the expected direction, and with impacts tending to be stronger for females. Table 5 shows the most important predictor among these measures is the Food Environment Index (FEI), a measure of food environment combining limited access to healthy foods with food insecurity. The impact of the FEI is much stronger for females, in line with survey based findings that food insecure adult women were more likely to be obese than food insecure males [73]. The effects of the two variables measuring exposure to fast food are both positive, while the impact of groceries per head is negative. The impact of the percent of local restaurants that are fast food is greater for women. Subject to the caveat that the present study is ecological, these findings are in line with research showing impacts of food access and security on obesity are mediated by gender [73,74]. 

## 5. Conclusions

A major area of ongoing environmental health research is focused on the effects of place on growing obesity levels [6]. In this paper, aspects of urban settlement and commuting, healthy food availability, and exercise access have been represented by seven area level indicators. Their impacts on geographic differences in obesity are assessed by regression methods that control for inter-correlations between indicators, and for spatially correlated residuals. The environmental indicators have mostly significant impacts on obesity, account for most of the poverty and regional effects on obesity, provide major increases in the proportion of explained variability, and account for much of the spatial clustering evident in obesity maps. The importance of activity levels/exercise access is supported, and also of settlement patterns (e.g., as expressed in residential density and car dependence). These conclusions have the advantage of being based on county data covering the entire US.

Limitations are, however, present. While the CDC county-level estimates data provide an entire national perspective they are subject to possible biases associated with the BRFSS, namely reliance on self-reported health status, and exclusion of households without phones. Another limitation concerns the available indicators of the environment [9,13]: for example, there is a direct measure available of inactivity levels at US county level, but not a direct measure of dietary quality, such as proportions eating five daily servings of fruit and vegetables. This may affect conclusions regarding the relative importance of activity and food environments. There is also a caveat regarding ecological studies, that one cannot make conclusions about individuals from an analysis of aggregate-level data [75].

As possible directions for further research, one may mention the need to extend the range of available measures of the food and activity environments, such as a direct county measure of dietary quality. It may also be useful to carry out regression analysis considering both obesity and related diseases (e.g., diabetes), to assess how far environmental impacts on these diseases are direct, or mediated by their impacts on obesity [76]. Geographical studies also have utility in indicating potential directions for population-based prevention and intervention, that is, not primarily targeted at the individual level or at particular high-risk groups. Such studies may be used to develop area profiles to identify communities where interventions, especially environmental interventions [77], may be most relevant. In particular, such interventions may include promotion of healthy eating environments, and promoting equity in physical activity, in line with a strategy to promote environmental justice with regard to obesity [67]. 

## Figures and Tables

**Table 1:** Obesity rates by urban-rural category and county poverty quintile.

Poverty Quintile *	Urban-rural Category **									
Males	1	2	3	4	5	6	7	8	9	All
1	28.0	30.4	30.5	29.0	27.6	31.7	30.3	31.9	31.3	29.9
2	29.8	29.8	30.4	31.5	28.5	31.9	31.3	30.6	31.5	30.8
3	30.6	30.6	31.4	32.3	33.1	31.6	30.6	30.9	31.1	31.2
4	29.3	31.5	31.8	32.1	31.3	32.8	32.1	32.7	31.1	31.8
5	31.3	32.5	30.9	32.2	32.7	33.5	33.4	33.4	33.5	33.0
All Counties	29.1	30.9	31.1	31.7	31.2	32.5	31.7	32.1	31.7	31.3
Females	1	2	3	4	5	6	7	8	9	All
1	25.9	28.4	27.9	27.0	24.9	28.9	27.7	28.7	27.9	27.4
2	28.6	28.3	28.4	29.4	26.3	29.9	29.0	28.2	28.9	28.8
3	30.1	29.6	30.4	30.8	31.6	30.1	28.7	28.8	28.8	29.8
4	30.1	31.8	31.5	31.3	30.5	32.1	31.0	32.3	29.5	31.3
5	36.0	33.5	32.4	33.6	36.0	35.2	34.5	36.1	33.9	34.5
All Counties	28.2	30.1	30.1	30.8	30.5	31.9	30.5	31.3	29.8	30.3

*: 1. 20% of counties with lowest poverty (under 11.8%); 2. Counties with poverty rates between 11.8% and 14.7%; 3. Counties with poverty rates between 14.7% and 18%; 4. Counties with poverty rates between 18% and 22.1%; 5. 20% of Counties with highest poverty (over 22.1%); **: 1. Counties in metro areas, 1 million population or more; 2. Counties in metro areas, 250,000 to 1 million population; 3. Counties in metro areas, fewer than 250,000 population; 4. Urban population, 20,000 or more, adjacent to a metro area; 5. Urban population, 20,000 or more, not adjacent to a metro area; 6. Urban population, 2500 to 19,999, adjacent to a metro area; 7. Urban population, 2500 to 19,999, not adjacent to a metro area; 8. Rural or less than 2500 urban population, adjacent to a metro area; 9. Rural or less than 2500 urban population, not adjacent to a metro area.

**Table 2:** Obesity rates by area poverty and majority race/ethnicity.

Males	Majority Race/Ethnic Category				
Poverty Quintile *	White N-H	Black N-H	Hispanic	Other	All
1	29.9	31.1	30.7	33.3	29.9
2	30.9	30.3	28.5	27.9	30.8
3	31.2	33.2	29.4	29.9	31.2
4	31.9	29.0	28.2	30.0	31.8
5	33.0	34.5	28.8	34.3	33.0
All counties	31.3	34.1	28.8	32.8	31.3
Females	Majority Race/Ethnic Category				
Poverty Quintile *	White N-H	Black N-H	Hispanic	Other	All
1	27.3	35.9	28.2	33.3	27.4
2	28.9	31.2	25.5	25.6	28.8
3	29.8	37.7	27.1	30.4	29.8
4	31.4	34.3	26.3	29.3	31.3
5	33.8	42.2	26.8	36.6	34.5
All counties	30.0	41.4	26.8	34.0	30.3

* Poverty quintile, see Table 1.

**Table 3:** Obesity rates by census division and county poverty quintile.

Poverty Quintile *	Census Division **									
Males	1	2	3	4	5	6	7	8	9	All
1	26.4	28.4	30.6	32.8	28.2	31.0	30.0	24.3	27.5	29.9
2	26.0	31.6	31.8	32.9	29.1	31.8	31.5	26.6	26.1	30.8
3	29.9	31.4	32.4	32.6	31.4	33.3	31.7	26.9	27.1	31.2
4	31.5	30.4	32.3	32.6	31.2	33.7	33.0	27.4	28.2	31.8
5	21.2	26.0	31.7	33.9	32.3	35.2	33.6	27.6	29.0	33.0
All counties	27.0	30.4	31.7	32.9	30.9	34.1	32.5	26.4	27.5	31.3
Females	1	2	3	4	5	6	7	8	9	All
1	23.4	24.5	29.3	29.2	27.2	30.3	27.1	22.0	26.4	27.4
2	24.4	28.5	30.7	30.0	28.5	31.6	29.3	24.2	24.8	28.8
3	29.9	28.2	31.3	30.7	31.0	32.8	29.8	24.6	26.0	29.8
4	31.0	27.1	31.7	31.4	31.7	34.1	31.7	25.5	27.6	31.3
5	22.3	28.9	31.7	34.0	35.1	38.4	33.1	27.1	28.1	34.5
All counties	25.0	27.1	30.7	30.2	31.7	35.5	31.0	24.4	26.5	30.3

*: See Table 1; **: 1. New England; 2. Middle Atlantic; 3. East North Central; 4. West North Central; 5. South Atlantic; 6. East South Central; 7. West South Central; 8. Mountain; 9. Pacific.

**Table 4:** Parameter estimates (posterior means, 95% credible intervals). Effects on obesity of geographic categories.

	Persons			Males			Females		
	Mean	2.5%	97.5%	Mean	2.5%	97.5%	Mean	2.5%	97.5%
% variation explained	33	29	38	28	21	33	38	32	43
% of residual variation spatially structured	63	59	68	64	59	69	65	61	70
Intercept	26.4	23.1	29.8	26.3	23.3	29.5	26.5	22.7	30.4
Urbanicity ^1^									
Metro counties, 250,000 to 1 million pop.	0.44	−1.29	1.82	0.74	−0.26	1.92	0.26	−1.06	1.48
Metro counties, fewer than 250,000 pop.	0.30	−1.18	1.40	0.61	−0.51	1.94	0.12	−1.38	1.58
Urban pop. >20,000, adjacent to metro area	1.10	−0.47	2.46	1.42	0.33	2.71	0.99	−0.35	2.41
Urban pop. >20,000, not adj. metro area	1.05	−0.49	2.56	1.27	0.04	2.78	0.94	−0.60	2.63
Urban pop., 2500 to 19,999, adj. metro area	1.17	−0.79	2.54	1.54	0.42	2.97	1.14	−0.25	2.89
Urban pop., 2500 to 19,999, not adj. metro area	0.43	−1.94	1.48	0.65	−0.83	1.64	0.08	−1.76	1.28
Rural or <2500 urban pop., adj. metro area	1.05	−0.15	2.65	1.34	0.23	2.70	0.98	−0.37	2.77
Rural or <2500 urban pop., not adj. metro area	0.38	−1.40	1.57	0.83	−0.24	1.98	0.05	−1.38	1.39
Census division ^2^									
Middle Atlantic	2.27	−0.75	5.14	3.64	1.26	6.22	1.31	−1.49	4.52
East North Central	2.32	−1.19	5.43	2.72	−0.50	5.71	2.08	−1.66	5.56
West North Central	3.52	0.05	6.65	4.55	1.23	7.75	2.32	−1.55	6.16
South Atlantic	1.75	−1.55	4.72	2.48	−0.31	5.28	0.98	−2.30	4.20
East South Central	4.37	0.71	7.24	4.70	1.66	7.77	3.97	0.33	7.61
West South Central	4.04	−0.05	7.16	5.59	2.62	8.77	2.24	−1.19	5.91
Mountain	−0.45	−4.11	3.07	0.13	−2.92	3.40	−0.99	−4.56	2.95
Pacific	−2.30	−6.36	1.81	−2.39	−5.73	1.19	−2.39	−6.18	1.66
County majority ethnicity/race ^3^									
Black N-H	2.66	1.59	3.82	0.72	−0.38	1.74	4.23	2.11	5.76
Hispanic	0.92	−0.21	2.35	0.90	−0.20	2.12	1.04	−0.49	2.96
Other	5.30	3.69	6.99	3.90	2.40	5.47	6.54	4.49	8.57
County poverty level ^4^									
Quintile 2	0.48	−1.21	1.30	0.33	−1.19	1.13	0.23	−3.06	1.67
Quintile 3	1.30	0.31	2.08	0.93	−0.21	1.68	1.45	−0.89	2.64
Quintile 4	1.65	0.56	2.48	0.98	−0.45	1.77	1.89	−0.77	3.21
Quintile 5	2.57	1.67	3.37	1.52	0.46	2.29	3.41	1.41	4.58
Variances first order interactions, geographic categories									
Urbanicity*division	0.021	0.003	0.084	0.028	0.003	0.109	0.021	0.003	0.088
Urbanicity*majority ethnic	0.256	0.004	1.612	0.227	0.004	0.912	0.362	0.004	1.477
Urbanicity*poverty status	0.073	0.006	0.205	0.056	0.005	0.169	0.104	0.009	0.286
Division*majority ethnic	0.094	0.003	0.649	0.078	0.003	0.472	0.175	0.003	1.289
Division*poverty status	0.129	0.005	0.409	0.120	0.007	0.369	0.189	0.013	0.549
Majority ethnic*poverty status	0.064	0.003	0.560	0.056	0.003	0.446	0.340	0.003	2.211

Reference category: Counties in metro areas, ^1^ million population or more; ^2^ Reference category: New England; ^3^ Reference category: Majority White N-H; ^4^ Reference category: Poverty Quintile 1. A*B denotes interaction between A and B.

**Table 5:** Parameter estimates (posterior means, 95% credible intervals). Regression including food environment, activity environment, concentration score.

	Persons			Males			Females		
	Mean	2.5%	97.5%	Mean	2.5%	97.5%	Mean	2.5%	97.5%
% variation explained	56	52	60	50	43	54	62	57	65
% of residual variation spatially structured	49	41	55	53	46	60	50	44	57
Intercept	25.1	22.4	27.8	24.3	21.6	26.9	25.4	22.0	28.7
Environmental indices									
Inactivity	15.2	14.2	16.3	12.8	11.9	13.8	18.0	16.8	19.1
Adequate exercise access	−0.98	−1.44	−0.50	−1.13	−1.56	−0.68	−0.8	−1.3	−0.3
Ratio fast food to grocery outlets	1.58	0.57	2.57	1.58	0.63	2.51	1.6	0.5	2.7
Groceries per head	−1.65	−3.36	0.06	−1.13	−2.75	0.45	−1.9	−3.8	0.0
Food environment index	−2.32	−3.40	−1.27	−0.68	−1.74	0.35	−3.9	−5.1	−2.7
% restaurants that are fast food	1.23	0.57	1.88	0.98	0.37	1.61	1.5	0.8	2.2
Concentration score	−16.5	−20.0	−13.1	−19.7	−23.0	−16.4	−13.8	−17.6	−9.9
Urbanicity ^1^									
Metro counties, 250,000 to 1 million pop.	0.16	−1.0	1.2	0.31	−0.51	1.25	−0.04	−1.27	1.06
Metro counties, fewer than 250,000 pop.	−0.14	−1.5	0.8	0.05	−0.95	0.89	−0.30	−1.65	0.75
Urban pop. >20,000, adjacent to metro area	0.41	−0.6	1.5	0.57	−0.34	1.85	0.34	−0.88	2.03
Urban pop. >20,000, not adj. metro area	0.52	−0.6	1.7	0.67	−0.23	1.79	0.43	−0.73	1.83
Urban pop., 2500 to 19,999, adj. metro area	0.48	−0.6	1.8	0.58	−0.29	1.56	0.40	−0.75	1.78
Urban pop., 2500 to 19,999, not adj. metro area	−0.17	−1.6	0.8	−0.02	−1.13	0.76	−0.38	−2.00	0.68
Rural or <2500 urban pop., adj. metro area	0.29	−0.8	1.5	0.41	−0.44	1.69	0.31	−0.82	2.02
Rural or <2500 urban pop., not adj. metro area	−0.14	−1.3	0.8	0.19	−0.86	1.07	−0.37	−1.93	0.72
Census division ^2^									
Middle Atlantic	0.46	−1.5	2.6	2.29	0.36	4.37	−0.60	−2.82	1.80
East North Central	−0.70	−3.0	1.9	0.52	−1.99	3.07	−0.76	−3.66	2.15
West North Central	0.21	−2.2	2.9	2.36	−0.09	5.07	−0.90	−3.72	2.19
South Atlantic	−0.47	−2.8	2.0	1.10	−1.41	3.51	−1.24	−4.09	1.54
East South Central	0.12	−2.2	2.6	1.74	−0.69	4.24	−0.68	−3.49	2.21
West South Central	−0.32	−2.8	2.2	2.49	0.16	5.26	−2.67	−5.38	0.55
Mountain	−3.40	−6.0	−0.5	−1.73	−4.36	1.01	−4.16	−7.15	−1.05
Pacific	−3.15	−5.9	0.3	−2.34	−4.98	0.68	−2.99	−6.04	0.47
County majority ethnicity/race ^3^									
Black N-H	2.25	1.2	3.1	0.72	−0.15	1.62	3.48	1.63	4.77
Hispanic	0.30	−0.6	1.3	0.41	−0.47	1.35	0.41	−0.80	1.79
Other	3.41	2.1	4.8	3.11	1.85	4.33	3.52	1.79	5.13
County poverty level ^4^									
Quintile 2	0.07	−1.1	0.7	0.09	−0.66	0.70	−0.39	−4.02	0.82
Quintile 3	0.44	−0.3	1.1	0.43	−0.26	0.99	0.18	−1.86	1.16
Quintile 4	0.57	−0.4	1.2	0.41	−0.54	1.02	0.33	−2.25	1.39
Quintile 5	1.04	0.3	1.7	0.69	0.02	1.31	1.09	−0.92	2.05
Variances first order interactions, geographic categories									
Urbanicity*division	0.011	0.002	0.033	0.011	0.003	0.040	0.013	0.003	0.046
Urbanicity*majority ethnic	0.164	0.004	0.813	0.137	0.004	0.655	0.258	0.004	1.120
Urbanicity*poverty status	0.036	0.004	0.122	0.018	0.003	0.060	0.068	0.005	0.205
Division*majority ethnic	0.033	0.003	0.216	0.031	0.003	0.164	0.048	0.003	0.340
Division poverty status	0.026	0.003	0.109	0.028	0.003	0.109	0.043	0.004	0.166
Majority ethnic*poverty status	0.043	0.003	0.287	0.037	0.003	0.218	0.272	0.003	1.919

^1^ Reference category: NCHS Category 1 (Counties in metro areas, 1 million population or more); ^2^ Reference category: New England; ^3^ Reference category: Majority White N-H; ^4^ Reference category: Poverty Quintile 1. A*B denotes interaction between A and B.
